# The Role of Bacterial Chaperones in the Circulative Transmission of Plant Viruses by Insect Vectors

**DOI:** 10.3390/v5061516

**Published:** 2013-06-19

**Authors:** Adi Kliot, Murad Ghanim

**Affiliations:** 1 Department of Entomology, The Volcani Center, Bet Dagan, 50250, Israel; E-Mail: adiaaa@gmail.com; 2 Institute of Plant Sciences and Genetics in Agriculture, Robert H. Smith Faculty of Agriculture, Food and Environment, Hebrew University of Jerusalem, POB 12, Rehovot, 76100, Israel

**Keywords:** *Bemisia tabaci*, GroEL, chaperone, virus, circulative transmission

## Abstract

Persistent circulative transmission of plant viruses involves complex interactions between the transmitted virus and its insect vector. Several studies have shown that insect vector proteins are involved in the passage and the transmission of the virus. Interestingly, proteins expressed by bacterial endosymbionts that reside in the insect vector, were also shown to influence the transmission of these viruses. Thus far, the transmission of two plant viruses that belong to different virus genera was shown to be facilitated by a bacterial chaperone protein called GroEL. This protein was shown to be implicated in the transmission of *Potato leafroll virus* (PLRV) by the green peach aphid *Myzus persicae*, and the transmission of *Tomato yellow leaf curl virus* (TYLCV) by the sweetpotato whitefly *Bemisia tabaci*. These tri-trophic levels of interactions and their possible evolutionary implications are reviewed.

## 1. Introduction — Circulative Transmission of Plant Viruses

It is widely accepted that the transmission of viruses to eukaryotic (animal and plant) hosts by arthropod vectors is coordinated between evolutionarily conserved, but highly specific, proteins. Arthropod-mediated transmission of each pathogen depends on a suite of well-coordinated interactions between evolutionarily conserved protein(s) encoded by the virus, vector and host. Viruses have evolved specific mechanisms to enable their transport across and within multiple vector/host tissues, and to evade or combat vector/host defenses. The specific types of molecules and the mechanisms that make possible such modes of virus transmission by arthropods are, however unstudied for the majority of insects and associated viral genera. This area is of particular interest as it has become more evident that the ability of a viral pathogen to be acquired and transmitted, and to evade host defenses is subject to adaptive, evolutionary change. 

Viruses that are vectored by arthropods exist in both the animal and the plant kingdoms. Plant viruses, can be generally categorized into two major groups based on their relationship with the vector; circulative and non-circulative. Circulative viruses are also termed ‘persistent’, and as more scientific evidence of their relations with their insect vector is gathered, this name is receiving more explanations for the nature of these virus-vector interactions. Circulative viruses belong to the *Luteoviridae*, *Geminiviridae* and *Nanoviridae* and are the most dependent upon their vector for successful transmission to new hosts and establishment of new infections. Most circulative plant viruses do not have the ability to move between plants and therefore mostly rely on their vector to be injected into the plant tissue from which they can develop a systemic infection in the xylem and, more often, the phloem. The majority of insects which are plant virus vectors are phloem feeders and all have sap sucking mouthparts [1]. Circulative plant viruses can be further divided into two sub-categories: Propagative and non-propagative. Propagative viruses are the most similar to animal viruses, some virus families include both members infecting animals and others infecting plants. Propagative viruses infect and reproduce in their plant host and insect vector, some causing deleterious effects to the vector [2,3,4]. This review article focuses on non-propagative viruses. A debate exists as to whether non-propagative viruses have some transcriptional activity in their insect vectors, as there is some evidence that transcripts of non-propagative viruses, such as begomoviruses, rise within the first few days following acquisition from the infected plant and retention in the vector [5]. Some begomoviruses, such as *Tomato yellow leaf curl virus* from Israel (TYLCV-IL) are retained for the entire whitefly life time, and are able to remain transmissible for a long time period after acquisition, some for the entire life span of the adult, indicating that a transmissible reservoir of the virus is retained at some unknown site in the insect [5,6,7,8,9]*.* TYLCV-IL has some other interesting and unexpected characteristics if compared to other TYLCV isolates. The Israeli isolate can be transferred from whitefly males to females and vice versa during mating [6]. TYLCV-IL can also be transovarially transmitted from viruliferous female to their next generation through the reproductive system [10]. *Squash leaf curl virus* (SLCV) is another whitefly-transmitted begomovirus that is not known to replicate in its whitefly vector. However, several studies have shown that this virus can reach or “infect” several whitefly tissues and organs, other than those implicated in the translocation route of the virus inside the insect body. SLCV was further shown to provoke reproductive problems in the whitefly vector; however such studies have never been thoroughly completed for further examining the molecular nature of these interactions, and further studies must be conducted. Begomoviruses are highly genetically diverse, even within different variants of the same virus isolates. This diversity, which leads to variations on the protein level, might explain some of the differences observed on the virus-vector interactions level [11,12,13].

All viruses that are transmitted in a circulative manner, as opposed to the non-circulative viruses, must undergo a latent time period within their vector in which they translocate. Latency occurs while translocating through various tissues along the transmission route in the insect, before the virus is injected into a new host plant. The path that the virus follows inside the insect, together with the organs crossed is generally similar for all circulative viruses transmitted in a circulative manner. The virus is acquired by the insect stylet while feeding from the plant phloem along with the ingested plant sap, similar to a blood meal ingested by a mosquito transmitting the malaria causative agent *Plasmodium*. The virus moves along the esophagus and enters the digestive system. Luteoviruses mainly cross the aphid hindgut to the hemolymph [14], while begomoviruses cross the midgut, specifically a specialized organ within the midgut called the filter chamber [6]. In the hemolymph, the virus circulates and reaches the salivary glands. While luteoviruses enter the accessory salivary glands of their aphid vectors [14], begomoviruses enter the primary salivary glands of whitefly vectors [6]. Viruses are emptied from the salivary glands through the salivary ducts into the salivary canal within the stylet. Few viruses, mainly propagative, infect other organs in the insect, other than those involved in the virus transmission, such as the reproductive system, and they can be vertically transmitted to the next generation [15]. The passage of several begomoviruses such as TYLCV-IL, SLCV, *Tomato mottle virus* (ToMoV) and *Cabbage leqf curl virus* (CaLCV) in *B. tabaci* using several light and electron microscopy techniques, as well as fluorescence *in situ* hybridization (FISH), immunogold and immune-flourescence methods have been studied and reviewed in detail in several research papers and reviews [6,16,17,18,19].

The circulative pathway for virus transmission described above involves crossing several barriers by the virus. Some of these barriers are cellular structures such as the gut and salivary gland membranes, while others are hostile environments for the virus, especially the hemolymph. Crossing the gut and the salivary glands involves entering epithelial cells, or infecting these cells by the virus, while avoiding attack by the immune system while circulating in the hemolymph. It has been previously shown that luteoviruses enter and exit the gut epithelial cells by endo- and exocytosis [11,14,20]. After crossing the gut epithelial cells, the virus must move and survive the hostile environment of the hemolymph, until entering the salivary glands. Previous studies postulated that if a virus is able to cross the gut and survive the insect hemolymph, it reaches a reservoir of transmissible virus particles that can survive and be transmitted by the insect for the rest of the vector’s life span [21]. While feeding, hemipterans secrete saliva used to facilitate the penetration of the insect’s stylet between and within the plant cells, and to combat plant defenses. Normally, virions leave the stylet and are injected specifically into their respective suitable plant tissue. In many cases, this tissue is the phloem, but can also be the xylem. Injecting plant viruses occurs along with salivary secretions [22]. The insect’s saliva is composed of two salivary secretions: watery saliva and gelling saliva. While the gelling saliva is thought to aid in the stylet’s penetration into the plant tissue, watery saliva is thought to include proteinous substances that aid in combating the plant defense system against insect feeding. Saliva secretion is also hypothesized to be a mechanism of disposing of certain materials traveling in the hemolymph, of which are included virus particles [15,23]. The Salivary Glands (SG) have a highly invaginated apical plasmalemma, strengthening this hypothesis [15,22]. It is possible that viruses employ receptors on the SG membrane, while the main function of these receptors is taking in materials from the hemolymph for secretion outside the insect. 

## 2. *Potato Leafroll Virus* (PLRV) Transmission by the Peach Potato Aphid *Myzus persicae* and the Coat Protein Role

*Potato leafroll virus* (PLRV) is the type member of the genus *Polerovirus* (family *Luteoviridae*) [24]. This virus replicates mainly in the phloem of host plants and is transmitted in a persistent-circulative manner by aphids, particularly *M. persicae* [25,26]. Generally, the latent period for luteoviruses can be as short as 24 hours; however it can also reach up to four days [14]. While early reports suggested that luteoviruses may replicate in their aphid vectors, this hypothesis was later rebutted, and it is now accepted that this group of viruses do not replicate in their aphid vectors [14,27,28]. The Coat Protein (CP) of plant viruses that depend on insect vectors for transmission, including luteoviruses has been shown to have an important role not only in both virus particle formation inside the plant and infection, but also in the aphid-mediated transmission [29]. Several studies have addressed the effects of mutations and structural changes in CP on virus aphid transmission. CP can influence several aspects of the aphid-virus interactions including the velocity of the virus passage through the aphid, but it also can influence the ability of the virus to flourish in the plant phloem [14]. Several trans-capsidation experiments verified the role of CP in determining the specific recognition between virus species and aphid vectors [30]. Virions that were assembled from CP alone were fed to aphids through artificial diet, and those virions reached the hemolymph, suggesting that the CP has all the necessary factors needed for movement through the gut cells [31,32,33]. The Read Through Domain (RTD), a constituent of the coat protein of luteoviruses, with unverified role, was shown to possess a role in BWYV translocation in the aphid body. Virus mutants that lacked the RTD crossed the aphid gut but with inferior efficiency, compared to wild type virus [14]. In addition, the transmission of two PLRV mutants in different RTD sequences was affected [25,34,35]. Research conducted using mutations in PLRV CP inferred that certain domains in the CP were necessary for proper transmission, and mostly included negatively charged amino acids [36]. The barrier conferring the most selective pressure on virus-vector specificity resides in the accessory salivary glands (ASG), as will be discussed later on in this review. All luteoviruses studied to date are transmitted via the ASG and not the Primary Salivary Gland (PSG) as is the case in whiteflies and begomoviruses [32,33,37,38]. PLRV particles have been observed specifically adhering to the ASG basal membrane, indicating recognition between the virus and possible receptors on the vector’s ASG. It is accepted that crossing the ASG membrane is possible via a receptor-mediated endocytosis/exocytosis mechanism [25,37].

## 3. *Tomato Yellow Leaf Curl Virus* (TYLCV) Transmission by the Sweetpotato Whitefly *Bemisia tabaci*

The phytophagous homopteran whitefly *Bemisia tabaci,* which specializes in plant vasculature feeding using slender mouthparts or stylets, transmits *Begomovirus* (Family: Geminiviridae), a plant virus group that belong to an emerging genus of ssDNA plant viruses. Begomoviruses exhibit a range of tissue tropisms in the plant with some being phloem-limited, and others not restricted to the phloem. In addition they are affiliated specifically with gut, hemolymph, and salivary organs of their whitefly vector, respectively, indicating evidence of cross-kingdom symbiosis with distinct co-adaptations to plant and animal hosts. Whether the whitefly vector derives direct benefit from this interaction is not known, but generally begomoviruses are not propagative in the vector, and so the extent of the symbiosis is not well understood. Even so, important clues suggest a possible degree of pathogenicity, or perhaps, parasitic relationship on the part of TYLCV with its B biotype whitefly vector [39,40], based on evidence of viral transcript accumulation following ingestion and acquisition [10,41,42], together with possible fitness benefits [43]. This is in contrast to other well-studied begomoviruses, which are not transcriptionally active in the whitefly, but are circulative, non-propagative and therefore have been considered ‘non-parasitic’, in the classical sense [44,45]. Indeed, supporting molecular, cellular, and functional explanations at all levels are lacking for these two phenotypic scenarios, and as well they are insightful implications to long-term virus-vector co-evolution. Perhaps TYLCV has made an important leap forward in exploiting new tissue tropisms, and may therefore be evolutionarily advancing in this respect compared to other extant begomoviruses. An added evolutionary conundrum holds that plant viruses that are propagative in their insect vector usually are thought to have arisen initially as parasites/pathogens of the insect, while later evolving the ability to exploit plant tissues that are fed upon by their insect host. Though TYLCV is not propagative, the above characteristics taken together with evidence of transovarial transmission (horizontal) [10], sets TYLCV apart from all other begomoviruses. Thus, it may well represent an intermediate type of begomovirus that presents a unique system for investigating the virus-vector co-adaptation. 

The whitefly vector of begomoviruses is comprised of all members of the *B. tabaci* sibling species group [46]; a suite of genetic and phenotypic variants comprising more than 30 biotypes and numerous haplotypes that are differentiated by DNA markers. *B. tabaci* biotypes differ genetically as well as in host range, insecticide-resistance and in ability to transmit plant viruses and to cause plant disorders. The most predominant and damaging biotypes are the B and Q biotypes [47,48,49,50,51,52]. While the B biotype is defined by extreme fitness in arid, irrigated cropping systems, the ability to efficiently transmit both New and Old World begomoviruses [47,48], causes phytotoxic-like symptoms due to feeding [53], the Q biotype is best known for its ability to develop resistance to certain insecticides, and to bewell-adapted to greenhouse environments [54]. Viral disease outbreaks over time have been mainly associated with the exotic B biotype, now widespread in locales where it is not endemic, suggesting it is highly fit and also a superior co-adapted vector for Old and New World begomoviruses. 

TYLCV infects only dicotyledonous plants and is transmitted only by the whitefly *B. tabaci* sibling species group [49,51]. Begomoviruses originating from the New World have bipartite genomes (SLCV), consisting of two components each ~2.6 kb. Five to six open reading frames encode viral proteins >10 kDa [55]. Begomoviruses all require the CP for encapsidation, certain movement functions, and for circulative transmission by the whitefly vector. The CP is the only viral-encoded protein required for vector-mediated transmission. Differences in competency is likely to be due to differences in co-adaptation between the virus and its vector haplotypes, perhaps evolving tighter binding affinity, avidity, or sequestration capacities to receptors or other protein effectors in the guts and/or salivary glands. For circulative TYLCV transmission, virions must be ingested and internalized by the vector. Virions must be transported across gut cellular membranes, occupy a temporary residence in the hemolymph, and then enter the salivary system [6,44,45]. TYLCV transmission requires an acquisition-access period (AAP) of 1–3 h on the infected plant, a latent period of 6–12 h during which the virus is not initially transmissible but becomes so following salivary gland entry, and a 30–60 min inoculation access period (IAP) on the host plant. Once acquired, virions are transmissible for the life of the whitefly [5,6]. The current model for the transmission pathway is based on the aphid-transmitted luteoviruses, *in vivo* transmission parameters, anatomical studies, and TEM localization [16,17,44,45,56]. In aphids and whiteflies, the gut membrane is thought to regulate transmission competency [16,57]. In this model, virions are transported into the cytoplasm in vesicles that fuse with the basal plasma membrane, releasing particles between the membrane and the basal lamina [44,45,56]. While the organ of specificity for luteoviruses is the ASG, for *B. tabaci* labeled TYLCV and SLCV has been observed only in the PSGs. A non-vector whitefly *Trialeurodes vaporariorum* (West.) has been shown to ingest begomoviruses, and in one instance evidence was provided for virus presence in the blood, however in neither instance did virus transmission occur indicating that vector specificity does not reside at the level of ingestion or at the gut interface [16,58]. Evidence points to the direct involvement of whitefly gut, salivary, and hemolymph- circulating whitefly proteins in begomovirus-whitefly circulative transmission [5,6]. In addition, one study has reported an abundant 60 kDa GroEL homologue with binding affinity to TYLCV virions [58]. GroEL was initially attributed to the primary symbiont, but another study has since suggested that the protein is encoded by the secondary endosymbiont, *Hamiltonella* [59]. Additionally, a whitefly-encoded HSP70 has been implicated in the circulative transmission of TYLCV, SLCV and WmCSV. This protein was shown to interact directly with TYLCV and SLCV [60], and to influence their transmission. 

## 4. Proteins Influencing the Circulative Transmission of Plant Viruses

As detailed above, different factors may influence the virus circulative transmission pathway within its insect vector. Some viruses transmitted in a non-circulative manner (both non-persistent and semipersistent) were shown to require Helper proteins to assist in their transmission [61]. Helper proteins are not always encoded by viral genes, and were implicated in the attachment of the virus to cuticular structures within the stylet and the insect foregut [61]. Such structures were described for only one virus transmitted by a circulative manner (FBNYV, [61]). This suggests that in circulative viruses, the specificity of the virus-vector interactions is mostly determined by the virus capsid, and not molecules that bridge the interaction with cuticular structures. In all viruses, however, even those that are not transmitted by a vector, they were shown to be acquired by an insect vector. Such is the case with many Luteoviruses; few cases were shown in which non-transmissible viruses where retained by a non-vector aphid. The degree of virus-vector specificity rises with each barrier that the virus must cross within the vector. Many viruses were located within gut epithelial cells of non-vector aphids, but none in the hemolymph and ASGs. Some were able to cross the gut and were detected in the hemolymph but not in the ASGs. In the hemolymph, the immune system functions as a barrier against viruses, and they are destroyed if no specificity resides in this tissue. Not much is known about the aphid immune system, and no hemocytes were found in the hemocoel of *M. persicae*, however, this might not be the case in all aphid species [22]. It was shown that different luteoviruses attach to different regions in the aphid digestive system; for example, while PLRV and BWYV were shown to cross the midgut to the hemolymph of *M. persicae*, the Soybean Dwarf Virus (SbDV) crosses at the hindgut. Several studies have concluded that in many luteovirus-aphid systems, the most selective barrier that regulates the transmission is the ASG cells. Recently, it was shown that ASG contain two barriers: the extracellular basal lamina wrapping the ASG cells and the basal plasmalemma of the ASG cells [62,63,64]. This implies that each membrane barrier requires a different receptor that recognizes different sites on the virus capsid with very high specificity [14,15]. Researchers have observed that when the aphid *S. avenae* acquired BYDV-MAV and later the serologically related BYDV-PAV, it transmitted the MAV variant more preferably than PAV [29,65]. When artificial mixtures of the two virus variants with different ratios were created, they noted that the higher the rate of MAV the lower the PAV transmission. When mixing PAV with MAV mutated CP, PAV transmission was uninterrupted. When PAV was mixed with another variant, PRV, both were efficiently transmitted. These results indicate that the BYDV variants MAV and PAV share a common CP domain and compete with each other on a binding site within the aphid, with the MAV variant having better binding affinity to this site. The site of this common receptor is yet unknown [29,65]. Studies aimed at the identification of aphid proteins that bind Luteoviruses revealed that BWYV binds to a protein in *M. persicae* whose homologue in *Drosophila melanogaster* was implicated in transcytosis [66]. BWYV particles were also bound to a membrane-GAPDH3 protein in the aphid. GAPDH3 is related to regulation of actin-dependent endo- and exocytosis in other organisms. This presents an example of the ability of one virus to bind to a few protein receptors in the aphid [66]. Two other proteins were also identified from the heads of aphids that transmit BYDV. The two proteins were not detected in heads of non-vector aphids [67]. In begomoviruses, the CP was shown to determine the insect-vector specificity [68,69,70,71,72]. The sequence of the CP in begmoviruses has very little variability, especially when compared to other proteins of this highly diverse virus family [11]. Two major sites in the CP of begomoviruses influence the specificity with relatively few amino acids [5,11]. When comparing the western hemisphere begomovirus SLCV transmission route and timing in the whitefly *B. tabaci*, with TYLCV, an eastern hemisphere begomovirus, it was concluded that as long as the virus is transmissible, its identity and the origin of the vector are irrelevant to the pathway the virus takes through [5,6]. In arboviruses, which infect animals, the insect’s saliva may contain substances that assist the virus in transmission to its host, a phenomenon termed Saliva Activated Transmission (SAT). Such factors are just now being discovered in the saliva of insect vectors of plant viruses, though it has been reported that aggregates of viruliferous aphids on a healthy host can form higher concentration of the virus in their feeding area than the rest of the plant [15,73,74,75,76,77]. On the other hand, no insect vector that has the ability to resist virus ingestion and/or transmission exists, although examples of viruses naturally losing their transmission ability have been described [70,71,78,79,80,81].

## 5. Bacterial GroEL Protein, Structure and Function, Including Symbiotic Bacteria of Insects

Several studies have shown that the process of protein folding in living cells is not a spontaneous procedure, and is mediated by a group of proteins termed ‘Chaperones’ [82]. These proteins, through the exploitation of ATP, help proteins fold into their final structure and are highly conserved from bacteria to all multi-cellular life forms. Of this group, *E. coli* GroEL and GroES are the most studied proteins; and named Chaperonins [83]. GroEL is an ATPas that appears in ring-shaped oligomers with or without GroES and is the major protein-folding assistant in bacterial cells [84,85]. When ATP and GroES bind to the GroEL ring with the protein ligand attached, a conformational change occurs that has not yet been fully mapped and is believed to change from one substrate to another [85,86,87]. Chaperonins are also used by many viruses that infect bacteria, animal or plants, in their virion assembly stage within the host cell, and thus it appears that GroEL proteins have the ability to bind proteins of different structures to fulfill their functional role in the folding or re-folding process [88]. Experiments examining *Tobacco Mosaic Virus* (TMV) assembly within *E. coli* cells showed that the levels of TMV dropped in cells without GroEL [82]. Complementary to these experiments, extremely high levels of TMV virions appeared in cells overproducing GroEL, emphasizing the role of the GroEL protein in proper TMV virion assembly [82]. A previous study on protein synthesis in the pea aphid *A. pisum* endosymbionts discovered ‘Symbionin’, a protein produced by the bacteria that is essential for proper symbiotic relations [89,90]. The same protein was later found in the primary endosymbiont of the green peach aphid *M. persicae*, *Buchnera*. The protein is highly homologous to both Symbionin and GroEL. In the *Buchnera* genome, the protein operon is split into two open reading frames with 72% and 73% nucleotide homology to GroES and GroEL respectively [91]. *In vivo*, the *Buchnera* GroEL is arranged like the GroEL from *E. coli* and arranged as an oligomer of 14 subunits forming two rings [92]. The *Buchnera* GroEL homologue from the sweetpotato whitefly *B. tabaci*, was later shown to be able to bind a variety of plant viruses, mediated by their capsid, mostly those whose virion is of globular or geminate shape, and whose CP has a high positive charge, a high percentage of arginine and a high isoelectric point—many of which are vectored by aphids, especially *M. persicae* [88]. In the next section we discuss two GroEL proteins that were described from bacterial endosymbionts of plant sap sucking insects: GroEL of *Buchnera*, the primary endosymbiont of aphids, and the GroEL of *Hamiltonella*, a secondary endosymbiont of *B. tabaci.* Both GroEL proteins were implicated in the transmission and survival of plant viruses while transmitted by their insect vectors [11,16]. 

## 6. Implication of GroEL Proteins in the Circulative Transmission of Aphid Transmitted Luteoviruses

A study by van den Heuvel *et al.* [89] performed a virus overlay assay in the search for proteins that interacted with PLRV virions or PLRV CP in *M. persicae* extracts. The study showed that out of five such proteins which interacted with anti-CP, four did not interact with the anti-idiotypic PLRV CP antibody (AiAb) that mimicked the surface structure of PLRV CP. One protein, however, did bind the AiAb and was the most abundant protein of the five identified in this screen [89]. This protein was highly homologous to a well-described protein from *E. coli* called GroEL. GroEL was previously termed ‘symbionin’ referring to its production by the endosymbionts of the pea aphid *A. pisum*. This protein was shown to be crucial for maintaining proper symbiotic relations between the aphid and the bacteria, and was found in all aphids including those that transmit PLRV. Immunogold labeling experiments using anti-GroEL antibody in *M. persicae* showed the localization of this protein within the cytoplasm of *Buchnera*, the primary endosymbiont of aphids. These endosymbionts reside in the hemolymph, within specialized organs named bacteriomes. GroEL was first termed MpSym (*M. persicae* symbionin) and later MpB GroEL (*M. persicae* Buchnera GroEL). Western blotting showed high levels of the protein in the aphids’ hemolymph. To ascertain whether this protein is associated with PLRV transmission through the aphid, aphids were fed with antibiotics. Although no change was recorded in the feeding behavior of the treated aphids, PLRV transmission was reduced by more than 70% [89]. Van den Heuvel *et al.* [89] later isolated GroEL proteins from endosymbitoc bacteria of three aphid species and *E. coli*, and tested their binding to various viruses of the luteovirus subgroup II and to viruses of other families that are aphid transmissible in a non-circulative manner. They showed that all luteoviruses bind GroEL proteins (with different affinities), even thosenon-transmissible by aphids. This affirmed the previous assays in which aphids were able to acquire viruses they did not transmit. None of the non-circulative viruses tested showed any affinity to the GroEL proteins. The fact that all luteoviruses bind to GroEL proteins suggested a common binding domain found in their capsid. Various mutant of BWYV were tested for their binding to MpB GroEL. Mutants in which the RTD in their CP was completely removed did not bind GroEL, however, mutants with deletion only in the C-terminal of RTD bound as efficiently as wild-type BWYV. This indicated that the conserved N-terminal of the RTD is the part of the luteovirus capsid required for GroEL binding. Levels of GroEL non-binding BWYV mutants in their RTD that were injected directly into the aphid hemolymph declined rapidly after the injection, suggesting an essential role for GroEL in virus survival in the hemolymph [92]. Further binding assays conducted with mutants of MpB GroEL and PLRV concluded that the protein binds the virus in a binding site located to both the C and N terminals of the GroEL protein, representing an area that was predicted by computer modeling as the equatorial domain of the protein in its tertiary structure [91]. When comparing the amino acid sequence of the PLRV binding site between MpSym and *E. coli* GroEL, this site was found to be conserved in all but one amino acid. Additional experiments conducted later characterized the nature of the PLRV-GroEL binding as hydrophilic. One conclusion that arose from these experiments was that the identity of the amino acids in the binding sites was not critical as long as the hydrophilic nature of the region was maintained [21]. 

## 7. Implication of the GroEL Protein in the Circulative Transmission of TYLCV

Many insects that are phloem-feeders such as the whitefly *B. tabaci* are known to harbor endosymbionts for completing their phloem unbalanced diet. *B. tabaci* harbors the obligatory primary endosymbiont like all whiteflies *Portiera aleyrodidarum*. Additionally, populations around the world were reported to harbor seven other secondary bacterial endosymbionts from different families including *Arsenophonus*, *Hamiltonella*, *Wolbachia*, *Cardinium*, *Fritschea*, *Rickettsia* and *Candidatus Hemipteriphilus asiaticus*) [93,94,95,96]. Those are facultative endosymbionts, termed also as secondary enosymbionts, with unknown function for the most part. It was found that different *B. tabaci* biotypes may harbor different secondary symbionts as was shown in the Israeli B and Q biotype populations [93]. Following the work conducted by van den Huevel *et al.*, in which a GroEL of *Buchnera* was implicated in the circulative transmission of PLRV through direct physical interaction [89], a similar GroEL protein was identified from the whitefy *B. tabaci* [97]*.* Testing the interaction between TYLCV and the GroEL homologue from *Buchnera* revealed a lack of interaction. This result itself was not surprising since *B. tabaci* is also known to harbor endosymbionts, and thus another GroEL from a different symbiont in *B. tabaci* might interact with TYLCV, if the same mechanism of virus transmission occurs in this whitefly-begomovirus system. *B. tabaci* transmits begomoviruses in a circulative manner, similar to that of luteoviruses in aphids. Following the studies conducted in aphids, Morin *et al.* identified a GroEL homologue to the *Buchnera* protein in the B biotype of *B. tabaci* from Israel. Whiteflies were fed with antibiotics or with anti-*Buchnera* GroEL antibodies and up to 80% reduction in TYLCV transmission was observed [97]. No TYLCV viral DNA was detected in the hemolymph of whiteflies fed with anti-GroEL antibodies prior to virus acquisition. The *B. tabaci* GroEL protein sequence share 80% homology with the *Buchnera* and *E. coli* GroELs [97]. These results indicated the involvement of GroEL from *B. tabaci* in TYLCV circulative transmission. Physical interaction between GroEL and TYLCV CP and virions, using western blot, yeast two-hybrid system and virus overlay assays, was then demonstrated [58]. Immunogold labeling on TEM thin sections from *B. tabaci* adults, using antibodies against the *Buchnera* GroEL showed that specific labeling was not associated with *Portiera*, the primary endosymbiont, but with another secondary symbiont. While the primary symbiont was termed “P”, referring to “primary” or “pleomorphic”, the secondary symbiont was termed “C” referring to “coccoid”. The specific immunogols labeling obtained in these experiments was associated with the “C” and not the “P” endosymbionts.

*B. tabaci* is characterized as a species complex, comprised of more than 30 different biotypes [46]. The B and Q biotypes are the most invasive and have a worldwide distribution [98,99]. In Israel, only the B and Q biotypes are present, and while the Israeli B transmits TYLCV with high efficiency, the Israeli Q biotype is considered a poor transmitter. Q biotype from Spain was reported as an efficient vector to TYLCV, even a better vector than the Spanish B biotype [59]. A survey of the secondary symbionts in Israel revealed that the B biotype was infected with *Hamiltonella* and *Rickettsia*, while the Q biotype was infected with *Rickettsia*, *Arsenophonus* and *Wolbachia* [93]. The Q biotype from Spain was also found to harbor *Hamiltonella*, in contrary to the Israeli Q biotype. A study conducted in Israel showed that the GroEL from *Hamiltonella* interacts with TYLCV CP in the Yeast two-hybrid system, immunocapture PCR and pull-down assays [60]. Other GroELs that were cloned from the other endosymbiotic bacteria from the B and Q biotypes did not interact with TYLCV CP [59,60]. These results suggest that the GroEL identified in the previous experiments conducted by Morin *et al.* [97] is a product of *Hamiltonella*. As in the case with the GroEL from *Buchnera*, the GroEL from *Hamiltonella* was described as an oligomer in the hemolymph of *B. tabaci,* but not in its digestive system [59]. One study demonstrated the ability of non-transmissible begomoviruses such as *Abutilon mosaic virus* from Israel (AbMV-Is) to interact with *B. tabaci* GroEL, similar to the *Buchnera* GroEL interaction with non-transmissible viruses by aphids [58]. Although in the aphid-luteovirus system, the PLRV RTD domain was implicated in the binding of *Buchnera* GroEL to PLRV CP, it is still likely that TYLCV binding to *Hamiltonella* GroEL is also based on hydrophilic nature, as is with the PLRV‑GroEL system [97].

**Figure 1:**
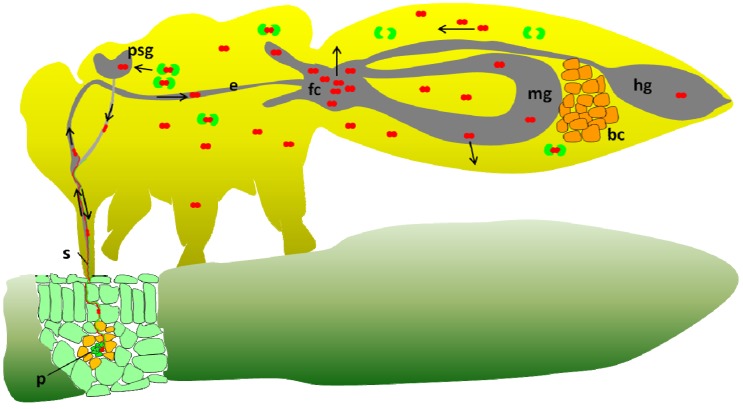
Existing model for *Tomato yellow leaf curl virus* (TYLCV) transmission by the B biotype of *B. tabaci*, and the involvement of *Hamiltonella* GroEL in the transmission process (this model is similar to the involvement of *Buchnera* GroEL from *M. persicae* in the transmission of *Potato leafroll virus* (PLRV)). p: phloem; s: stylet; e: esophagus; fc: filter chamber; mg: midgut; h: hindgut; psg: primary salivary glands; bc: bacteriocytes; green particles: GroEL; red particles: TYLCV virions; blue arrow: passage of TYLCV virion from the midgut in the thorax to the psg; black arrows: route of TYLCV while translocating in the whitefly. For more details see the text.

**Figure 2:**
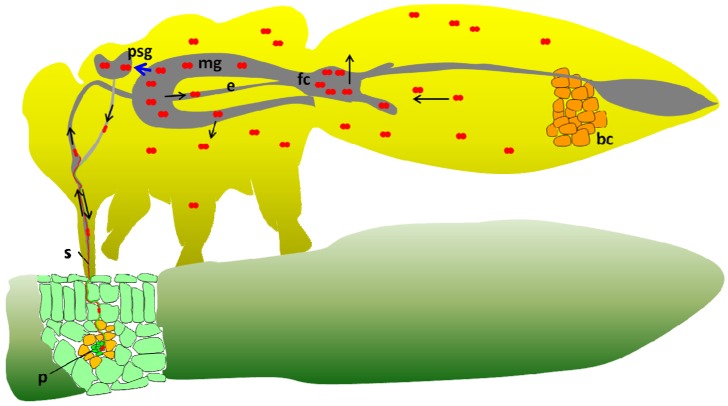
A first model for TYLCV transmission by the Q biotype lacking the *Hamiltonella* GroEL protein for proper transmission. Note the presence of the midgut in the thorax after being pushed by the ovaries in gravid females. See the legend for Figure 1 for abbreviations, and the main text for more details.

**Figure 3:**
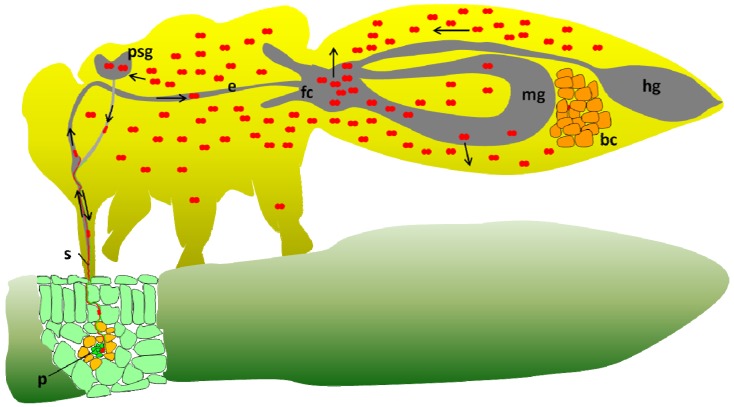
A second model for TYLCV transmission by the Q biotype lacking the *Hamiltonella* GroEL protein for proper transmission. Note the high concentration of TYLCV acquired by the whitefly. See the legend for Figure 1 for abbreviations, and the main text for more details.

One question that remains unanswered in the *B. tabaci-*begomovirus system is the ability of the Q biotype that lacks *Hamiltonella* (from Israel) to transmit TYLCV. Although this Q biotype is a poor transmitter, its ability to transmit the virus suggests that TYLCV is able to overcome the hostile environment within the insect’s hemolymph, where the protection by GroEL is provided. The lack of *Hamiltonella* further suggests that the relevant GroEL required for proper circulation and transmission of TYLCV in the Q biotype is absent. No direct evidence exists to suggest a proper explanation; however, several hypotheses can be raised and are detailed in Figure 1. Figure 1 presents the existing model for TYLCV transmission by *B. tabaci* B biotype, which in many extents is similar to the model that exists for the aphid-luteovirus system*.* The transmission cycle starts by virus acquisition from the phloem of an infected plant, the virus moves along the stylet, forgut, esophagus and reaches the midgut in whiteflies and further the hindgut in some aphid-luteovirus systems. In whiteflies, the majority of the virus particles are absorbed in the filter chamber to the hemolymph while some circulate in the midgut and are absorbed along the midgut loop (Figure 1). In aphids, viruses are absorbed in the hindgut and some in the midgut. In the hemolymph, TYLCV particles in *B. tabaci* and PLRV in *M. persicae* interact with the GroEL proteins produced by *Hamiltonella* in *B. tabaci* and by *Buchnera* in *M. persicae*, and are safeguarded until reaching the basal lamina of the primary salivary glands (PSG). TYLCV and PLRV virions attach to unknown receptors on the PSG and ASG membranes respectively, and are internalized into the glands cells, are then emptied into the glands main lumen which connects to the salivary canal in the stylet. From this canal virions are injected into the plant with salivary secretions. Figure 2 presents one possible explanation for the ability of the Q biotype of *B. tabaci* to transmit TYLCV. In this model, the midgut is pushed into the thorax of viruliferous adults, especially gravid females, when the abdomen is filled with developing eggs. Such a scenario brings the midgut very close to the PSG and significantly shortens the distance that TYLCV virions have to make before entering the gland cells (blue arrow in the figure). Such a scenario enables the virus to avoid the hostile environment in the hemolymph and rapidly enter the PSG, when protection is not available by GroEL. Pushing the midgut into the thorax of gravid females was shown to increase with age, suggesting that the more the insect is filled with eggs, the higher the chance for the midgut to be pushed into the thorax [100]. Figure 3 presents an additional model for the ability of the Q biotype to transmit TYLCV. In this model it is hypothesized that since whiteflies acquire varying amounts of the virus from infected plants, some insects acquire very high levels of the virus [18]. In such a situation, there is higher probability that enough virus particles will escape the hostile environment in the hemolymph without being destroyed, reach and enter the PSG and be transmitted. While such situations in the models presented in Figure 2, Figure 3 may occur, their chances are low, as expressed by the low ability of the Q biotype to transmit the virus. Gottlieb *et al.* [59] reported that while the ability of B biotype populations to transmit TYLCV reaches as high as 80% in one insect per plant transmission tests, the ability of Q biotype populations may only reach 5%, and can be higher if the two scenarios presented in Figure 2, Figure 3 occur together. However, the ability of the B biotype to transmit TYLCV is far higher, if compared with the Q biotype [60]. 

## 8. Conclusions

We reviewed the factors influencing circulative plant virus transmission by arthropod vectors and focused on the aphid–luteovirus and the whitefly–begomovirus systems. Three major sites in the insect regulate virus transmission. Those sites include the gut, hemolymph and the salivary glands. In the gut and salivary gland tissues, several insect proteins, such as specific receptors that interact with the virus CP, are expected to be involved and extensive research is ongoing to identify such proteins. In the hemolymph, the transmitted virus is exposed to attack by the insect immune system, and the lack of protection in the hemolymph is expected to significantly influence the successful transmission of the virus. Indeed, it has been shown in the *M. persicae*–PLRV and *B. tabaci*–TYLCV systems that endosymbiotic bacteria produce a GroEL protein that interacts with the transmitted virus in the hemolymph. This interaction provides proper protection for the virus and increases its probability to be transmitted. In the *M. persicae*–PLRV system, GroEL is produced by the primary endosymbint *Buchnera.* Primary endosymbionts are essential for the species persistence and complete the diet of their insect host, while the host provides the necessary protected environment for the bacteria. In the *B. tabaci*–TYLCV system however, GroEL is produced by a secondary endosymbiont that is not essential for the insect. An important question that arises in the *B. tabaci*–TYLCV system is which benefits are gained upon GroEL production by *Hamiltonella*, which do not provide any substantial benefit for the whitefly? Several lines of evidence lately suggested that circulative viruses may resemble propagative viruses that negatively influence the biology of the insect. This is most evident for TYLCV that has been shown to influence life span parameters of its whitefly vector including mortality, fecundity and fertility, and has been further shown to significantly alter global gene expression in the whitefly [39,60,101]. Exploitation the GroEL protein produced by these endosymbiotic bacteria might be a mechanism to overcome the negative effects of circulative viruses. This is obtained by specifically interacting with these viruses, bringing them to minimal interaction with insect tissues and thus avoiding their possible negative effect. This evolutionary adaptation works also in the favor of the virus, which can be efficiently and rapidly transmitted to the plant, a more suitable environment for its replication and further spread. 
